# High magnitude of carbapenemase-producing *Acinetobacter baumannii* in sepsis patients at Ethiopian referral hospitals: a whole genome analysis

**DOI:** 10.1038/s41598-026-44498-1

**Published:** 2026-03-18

**Authors:** Melese Hailu Legese, Daniel Asrat, Adane Mihret, Badrul Hasan, Abraham Aseffa, Göte Swedberg

**Affiliations:** 1 Department of Medical Laboratory Sciences, College of Health Sciences, Addis Ababa University, Addis Ababa, Ethiopia; 2 Armauer Hansen Research Institute, Addis Ababa, Ethiopia; 3 Department of Medical Biochemistry and Microbiology, Biomedical Centre, Uppsala University, Uppsala, Sweden; 4 Department of Microbiology, Immunology and Parasitology, College of Health Sciences, Addis Ababa University, Addis Ababa, Ethiopia

**Keywords:** Sepsis, Carbapenemase-producing *A. baumannii*, Antimicrobial resistance genes, Sequence types, Whole genome sequencing, Ethiopia, Diseases, Medical research, Microbiology

## Abstract

Carbapenemase-producing *Acinetobacter baumannii* (CP-Ab) is a critical priority pathogen. Between October 2019 and September 2020, a multicentre study was conducted at four Ethiopian hospitals: Tikur Anbessa and Yekatit (central), Hawassa (southern), and Dessie (northern). A total of 1416 sepsis patients were enrolled, and blood cultures were performed. *Acinetobacter* isolates were confirmed using MALDI-TOF and tested for carbapenem susceptibility. All *Acinetobacter* isolates were subjected to whole-genome sequencing. Selected isolates underwent nanopore sequencing through Plasmidsaurus. Forty-five *Acinetobacter* isolates were identified, mostly *A. baumannii* (*n* = 38), with a few other species (*n* = 7). Among the 38 *A. baumannii* isolates, 18 carried *bla*_NDM−1_ and either *bla*_OXA−23_ or *bla*_OXA−58_ carbapenemase genes concurrently. *bla*_OXA−58_ and *bla*_NDM−1_ were co-located on plasmids of sizes 80 kb to 113 kb. *bla*_OXA−66_ (*n* = 13) and *bla*_OXA−69_ (*n* = 12) were frequently identified chromosomally encoded carbapenemase genes. Several STs of *A. baumannii* were identified, with ST2 (*n* = 14) and ST1 (*n* = 13) being frequent. One *A. nosocomialis* carried *bla*_NDM−1_ and *bla*_OXA−58_ simultaneously. Several other genes were identified that confer resistance to aminoglycosides (*n* = 37), phenicol (*n* = 19), trimethoprim (*n* = 16), macrolides (*n* = 25), quinolones (*n* = 10), tetracyclines (*n* = 22), sulphonamides (*n* = 36), and disinfectants (*n* = 23). The high prevalence of carbapenemase-producing *A. baumannii* and other *Acinetobacter* species underscores the need for nationwide antibiotic stewardship.

**Supplementary Information:**

The online version contains supplementary material available at 10.1038/s41598-026-44498-1.

## Introduction

Globally, *A. baumannii* has emerged as a highly problematic pathogen, causing serious infections^1^. It is a significant cause of sepsis^2^, which is a life-threatening medical condition associated with important biological and chemical abnormalities, with a high mortality rate^3^. Managing sepsis caused by *A. baumannii* is tremendously challenging because of its resistance to virtually all available antibiotics, including carbapenems, the last-line treatment option for *Acinetobacter* infections. The main mechanisms of antibiotic resistance in *A. baumannii* include biofilm formation, reduced membrane permeability, antibiotic efflux, and acquisition of drug resistance via mobile genetic elements^4–7^.

The World Health Organization (WHO) identified carbapenem-resistant *A. baumannii* as a critical priority pathogen in 2017^8^ and reiterated this classification in 2024^9^. The main mechanism of carbapenem resistance is the production of carbapenem-hydrolyzing enzymes, known as carbapenemases^10^. These enzymes are typically of OXA-type, class D, and class B ^11^. The most problematic carbapenemase genes include *bla*_OXA−23_, *bla*_OXA−25_, *bla*_OXA−26_, *bla*_OXA−40_, *bla*_OXA−49,_*bla*_OXA−58,_ and *bla*_NDM−1_^7,11,12^.

Other *Acinetobacter* species (*A. nosocomialis*,* A. johnsonii*, *A. haemolyticus*,* A. radioresistens*,* A. lwoffii*,* and A. bereziniae)* harboring OXA-type carbapenemase genes have also been reported in hospital settings, causing serious infections^12,13^.

Data on the molecular epidemiology of carbapenemase-producing *A. baumannii* in sub-Saharan Africa, including Ethiopia, are inadequate. Moreover, the carbapenemase carriage rate among other *Acinetobacter* species is poorly defined in Ethiopia. Hence, this study aimed to determine the molecular epidemiology of carbapenemase-producing *Acinetobacter* species among sepsis patients enrolled at four hospitals in the central, southern, and northern regions of Ethiopia. A whole-genome analysis was employed to accurately determine the molecular features of *A. baumannii* and other *Acinetobacter* species.

## Materials and methods

This study presents a cross-sectional analysis of a prospective cohort comprising cases from October 2019 to September 2020, selected from hospitals in different regions of Ethiopia. The selection criteria prioritized universities and/or referral hospitals with established microbiology laboratories or connections to nearby government regional microbiology laboratories. Four Ethiopian universities and their associated referral hospitals from the central, southern, and northern regions were included in the study. The selected hospitals are Tikur Anbessa Specialized Hospital (TASH) and Yekatit 12 Specialized Hospital Medical College (Y12HMC) in the central region, Hawassa University Comprehensive Specialized Hospital (HUCSH) in the southern region, and Dessie Referral Hospital (DRH) in the northern region.

Patients diagnosed with sepsis through clinical evaluation in all hospitals were included in this study. Attending physicians diagnosed sepsis based on at least two of the following signs: abnormal body temperature (> 38 °C or < 36 °C), rapid heartbeat (heart rate > 90 bpm), rapid breathing (respiratory rate > 20 breaths/min), PaCO₂ below 32 mm Hg, and abnormal white blood cell counts (WBC > 12,000/mm³ or < 4,000/mm³). All age groups and both genders were included. However, patients who had received antibiotics within the past 10 days were excluded. Data on patient sociodemographic and clinical characteristics were collected. Details of the study are available in a previously published paper^14^.

### Blood culture and *Acinetobacter* isolation

A total of 1,416 patients diagnosed with sepsis across all study sites were enrolled, and each patient had one blood culture bottle processed to isolate *Acinetobacter* species. Of all patients investigated for sepsis, 45 *Acinetobacter* species were isolated, while the remainder were other bacterial species as described in the previous paper^14^. *Acinetobacter* species were phenotypically identified and characterized using colony morphology, Gram staining, and conventional biochemical tests. Biochemical media such as indole, urea, citrate, triple sugar iron, lysine decarboxylase, motility, and oxidase were used to characterize *Acinetobacter* species. After phenotypic characterization, all *Acinetobacter* strains were stored at -70 °C or -16 °C until they were transferred to Uppsala University in Sweden for further identification and characterization using advanced technologies.

### Re-identification of *Acinetobacter* species using MALDI-TOF

For confirmation, all *Acinetobacter* isolates were retyped using MALDI-TOF (Bruker Daltonics GmbH, Bremen, Germany) according to the manufacturer’s guidelines at the Clinical Microbiology Departments of Uppsala University Hospital and Karolinska Institute, Sweden. All isolates were refreshed on MacConkey or nutrient agar, and a single colony was smeared onto a MALDI-TOF plate and air-dried. Then, 1 µL formic acid was added to each cell, which was air-dried, and 1 µL MALDI matrix solution was applied to the cells. The plates were ready for reading after air drying. MALDI-TOF identification was automatically scored by the system software between 1 and 3 points, and all isolates scoring 2 or higher were accepted.

### Antimicrobial susceptibility testing of *Acinetobacter* isolates

Antimicrobial susceptibility testing (AST) was conducted using disk diffusion and interpreted according to the standardized table published by the Clinical and Laboratory Standards Institute (CLSI)^15^. Each *Acinetobacter* isolate was tested against ampicillin-sulbactam (10/10 µg), piperacillin-tazobactam (100/10 µg), cefepime (30 µg), cefotaxime (30 µg), ceftriaxone (30 µg), ceftazidime (30 µg), ciprofloxacin (5 µg), doxycycline (30 µg), gentamicin (10 µg), imipenem (10 µg), meropenem (10 µg), and Trimethoprim-Sulfamethoxazole (SXT) (1.25 /23.75 µg).

### Carbapenem susceptibility testing using broth dilution and E-Test strip

All Acinetobacter isolates were tested for meropenem and imipenem Minimum Inhibitory Concentration (MIC) using E-Test strips and broth dilution. The broth dilution test was conducted according to the CLSI guideline^15^, while the E-Test strip was performed following the manufacturer’s instructions. A cut-off value from CLSI^15^ was used to interpret broth dilution and E-test MIC results as sensitive, intermediate, or resistant to meropenem and imipenem. The MIC was defined as the lowest drug concentration that completely inhibited visible growth.

### Cefiderocol susceptibility testing using broth dilution and E-Test strip

All *bla*_NDM−1_-carrying *Acinetobacter* isolates were further tested for cefiderocol susceptibility using broth dilution according to the CLSI guidelines^15^, and the E-Test strip was performed following the manufacturer’s instructions. For broth microdilution testing, the ComASP cefiderocol 0.008–256 µg/ml broth microdilution test panel from Liofilchem, Roseto degli Abruzzi, Italy, was utilized. Bacteria were suspended in physiological NaCl to a 0.5 McFarland standard and mixed with iron-depleted, cation-adjusted Müller-Hinton broth. 100 µl of the bacterial suspension was added to each well of the test plates. The plates were incubated at 37 °C for 20 h.

### DNA extraction and whole genome sequencing (WGS)

The QIAamp DNA Mini Kit (QIAGEN, Germany) was used to manually extract DNA, and the Qubit™ 3.0 (Thermo Scientific, MA, USA) was used to measure DNA concentration. At the Science for Life Laboratory in Solna, Sweden, WGS was performed after transferring 20 µL of each DNA sample into a 96-well WGS plate (average 10 ng). Sequencing libraries were prepared using Nextera XT (Illumina) kits, and short-read sequencing was performed on Illumina HiSeq 2500 systems with a 150-bp paired-end protocol at the same facility. Coverage was generally just above 100%, with some samples below 100%. For a selected group of isolates, bacterial genome sequencing was performed by Plasmidsaurus (San Francisco, CA) using Oxford Nanopore Technology, with custom analysis and annotation. The coverage in these cases was high, around 100X.

### Whole genome sequencing of *Acinetobacter* isolates and bioinformatic analyses

SPAdes (version 3.9) was used for genome assembly. Using the assembled genomes, tools available at the National Center for Biotechnology Information (NCBI) were used for genome analysis. A Basic Local Alignment Search (BLAST) for all *Acinetobacter* isolates was performed with BLASTN version 2.13.0 + at https://blast.ncbi.nlm.nih.gov/Blast.cgi. Carbapenem resistance and other AMR genes present in each *Acinetobacter* isolate were identified using ResFinder 4.6, available at https://www.genomicepidemiology.org/services/. The phylogenetic tree of all *Acinetobacter* isolates was constructed using CSI Phylogeny 1.4 at https://cge.cbs.dtu.dk/services/CSIPhylogeny/, and the tree and metadata were visualized with iTOL version 6.5.2 at https://itol.embl.de/userInfo.cgi. Sequence Types of *A. baumannii* were determined using MLST version 2.0 at https://cge.food.dtu.dk/services/MLST/.

The Addis Ababa University, Armauer Hansen Research Institute, and Uppsala University approved all experiments performed in this study. All experiments were performed in accordance with relevant guidelines and regulations, which are cited above under each experiment section.

## Results

### *Acinetobacter* species: identification and frequencies

**Fig. 1:**
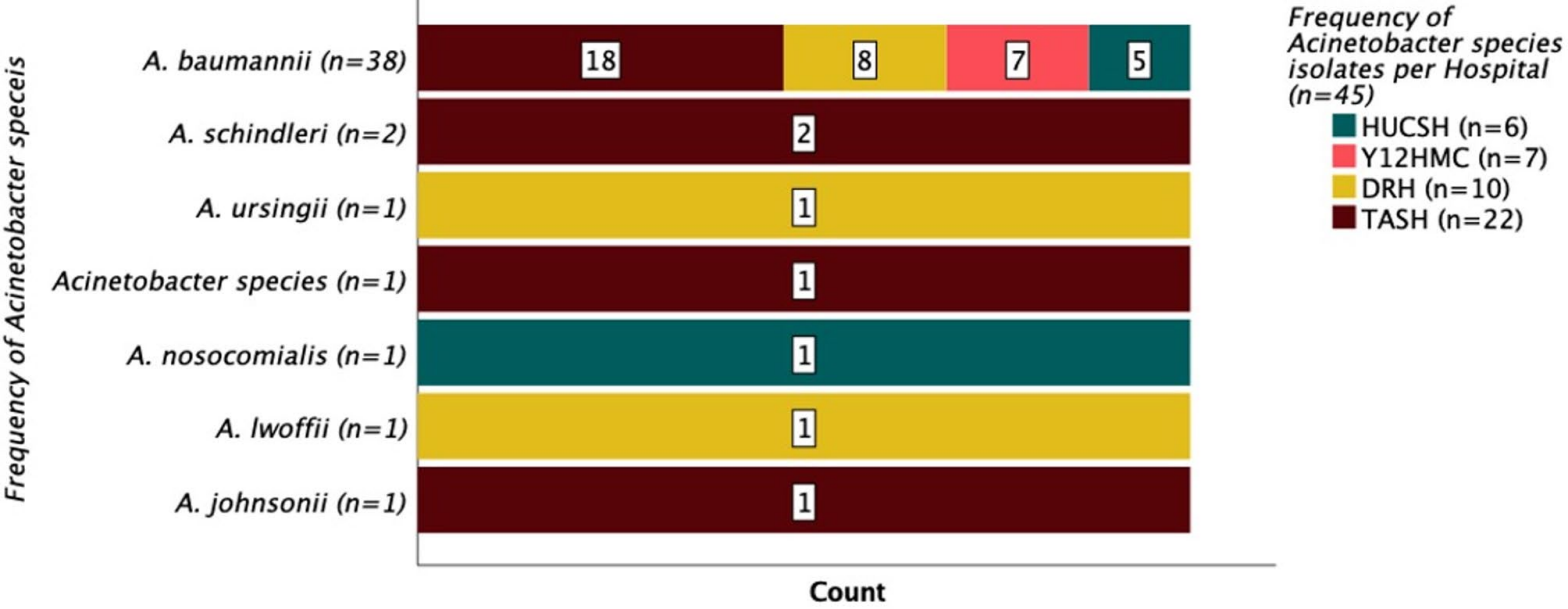
Frequencies and distributions of Acinetobacter species across the four hospitals. TASH – Tikur Anbessa Specialized Hospital; Y12HMC – Yekatit 12 Specialized Hospital Medical College; DRH – Dessie Referral Hospital, HUCSH – Hawassa University Comprehensive Specialized Hospital.

The present study identified 45 *Acinetobacter* species among 1416 patients evaluated for sepsis at TASH, Y12HMC, DRH, and HUCSH. Nearly half of the *Acinetobacter* isolates (*n* = 22) were found at TASH, while the isolation rates at DRH, Y12HMC, and HUCSH were 10, 7, and 6, respectively. *A. baumannii* (*n* = 38) was the most common species, with most (*n* = 18) identified at TASH (Fig. 1).

All Acinetobacter isolates were identified in hospitalized patients (Table 1). Most were found in the NICU (*n* = 22) and pediatric departments (Table 1), while nine isolates came from adult patients (over 18 years old) (Table 1). Most Acinetobacter isolates were from patients hospitalized for 1 week (*n* = 22), followed by those hospitalized longer than 4 weeks (*n* = 12). The majority (*n* = 31) of isolates were from patients referred from other health facilities for better care (Table 1).

**Table 1:** Frequency of *Acinetobacter* species in relation to patient sociodemographic characteristics.

Patient characteristics		Frequency of *Acinetobacter* species isolated (*N* = 45)	
		No.	Percentage (%)
Hospital	TASH	22	49
	DRH	10	22
	Y12HMC	7	16
	HUCSH	6	13
Admission status	Inpatient	45	100
	Outpatient	0	0
Gender	Male	21	47
	Female	24	53
Age category	≤29 days	22	49
	30 days − ≤1 year	4	9
	> 1–≤5 year	4	9
	> 5 - <18 year	6	13
	≥18 years	9	20
Ward	NICU	22	49
	EOPD	2	4
	Medical Ward	4	9
	Paediatrics	14	31
	Surgical	3	7
Hospital stay duration	1 week	22	49
	2 weeks	7	16
	3 weeks and above	4	9
	4 weeks and above	12	26
Underlying diseases	Yes	24	53
	No	21	47
Previous hospitalization	Yes	10	22
	No	35	78
Referral patient*	Yes	31	69
	No	14	31

TASH – Tikur Anbessa Specialized Hospital; Y12HMC – Yekatit 12 Specialized Hospital Medical College; DRH – Dessie Referral Hospital, HUCSH – Hawassa University Comprehensive Specialized Hospital; NICU- Neonatal Intensive Care Unit; EOPD- Emergency Outpatient Department * Patients who were transferred from other healthcare facilities to the study sites.

### Antimicrobial susceptibility profiles of *A. baumannii* and other *Acinetobacter* species

Most *A. baumannii* isolates showed resistance to cephalosporins, including cefepime, cefotaxime, ceftazidime, and ceftriaxone. Using disk diffusion, broth dilution, and E-test methods, the majority of these isolates were resistant to carbapenems, particularly meropenem (Table 2 & Supplementary Table 1). All *A. baumannii* isolates were multidrug-resistant, with six identified as pan-drug-resistant (Supplementary Table 1). Resistance to doxycycline and ciprofloxacin was less common. Although less frequently found, other *Acinetobacter* species, such as *A. johnsonii*, *A. nosocomialis*, *A. schindleri*, and related species, also exhibited resistance to carbapenems across disk diffusion, broth MIC, and E-test tests (Table 2). A subset of isolates, including all *bla*_NDM−1_ carriers, was tested for susceptibility to cefiderocol. While MICs ranged from 0.024 to 2.5 µg/ml, all tested *Acinetobacter* species were susceptible to cefiderocol per CLSI standards, with no resistance detected.

**Table 2:** Antimicrobial resistance patterns of *Acinetobacter* isolates identified from sepsis patients in Ethiopian referral hospitals.

	Antibiotics tested	*A. baumannii* (*N* = 38) *n* %	*A. johnsonii* (*N*= 1) *n* %	*A. lwoffii* (*N*= 1) *n* %	*A. nosocomialis* (*N*= 1) *n* %	*A. schindleri* (*N* = 2) *n* %	*Acinetobacter species* (*N* = 1) *n* %	*A. ursingii* (*N* = 1) *n* %
Disk diffusion	Ampicillin-sulbactam	28(74)	-	-	-	0(0)	1(100)	0(0)
	piperacillin-tazobactam	29(76)	-	-	-	0(0)	0(0)	0(0)
	cefepime	35(92)	1(100)	-	1(100)	1(50)	1(100)	1(100)
	cefotaxime	37(97)	1(100)	-	1(100)	2(100)	1(100)	1(100)
	ceftriaxone	38(100)	1(100)	1(100)	1(100)	2(100)	1(100)	1(100)
	ceftazidime	36(95)	1(100)	-	1(100)	1(50)	1(100)	1(100)
	ciprofloxacin	17(45)	-	-	1(100)	0(0)	0(0)	0(0)
	doxycycline	13(34)	-	-	-	0(0)	0(0)	0(0)
	gentamicin	26(68)	-	-	1(100)	0(0)	1(100)	0(0)
	meropenem	33(87)	1(100)	-	1(100)	1(50)	1(100)	0(0)
E-test (Strip-MIC)	Meropenem	29(76)	0(0)	0(0)	1(100)	1(50)	0(0)	0(0)
	Imipenem	22(58)	0(0)	0(0)	1(100)	0(0)	0(0)	0(0)
Microbroth dilution-MIC	Meropenem	30(79)	1(100)	0(0)	1(100)	1(50)	1(100)	0(0)

n- number of isolates; %- percentage per specific isolate.

### Carriage of carbapenemase-encoding genes among *A. baumannii* and other *Acinetobacter* species

**Fig. 2:**
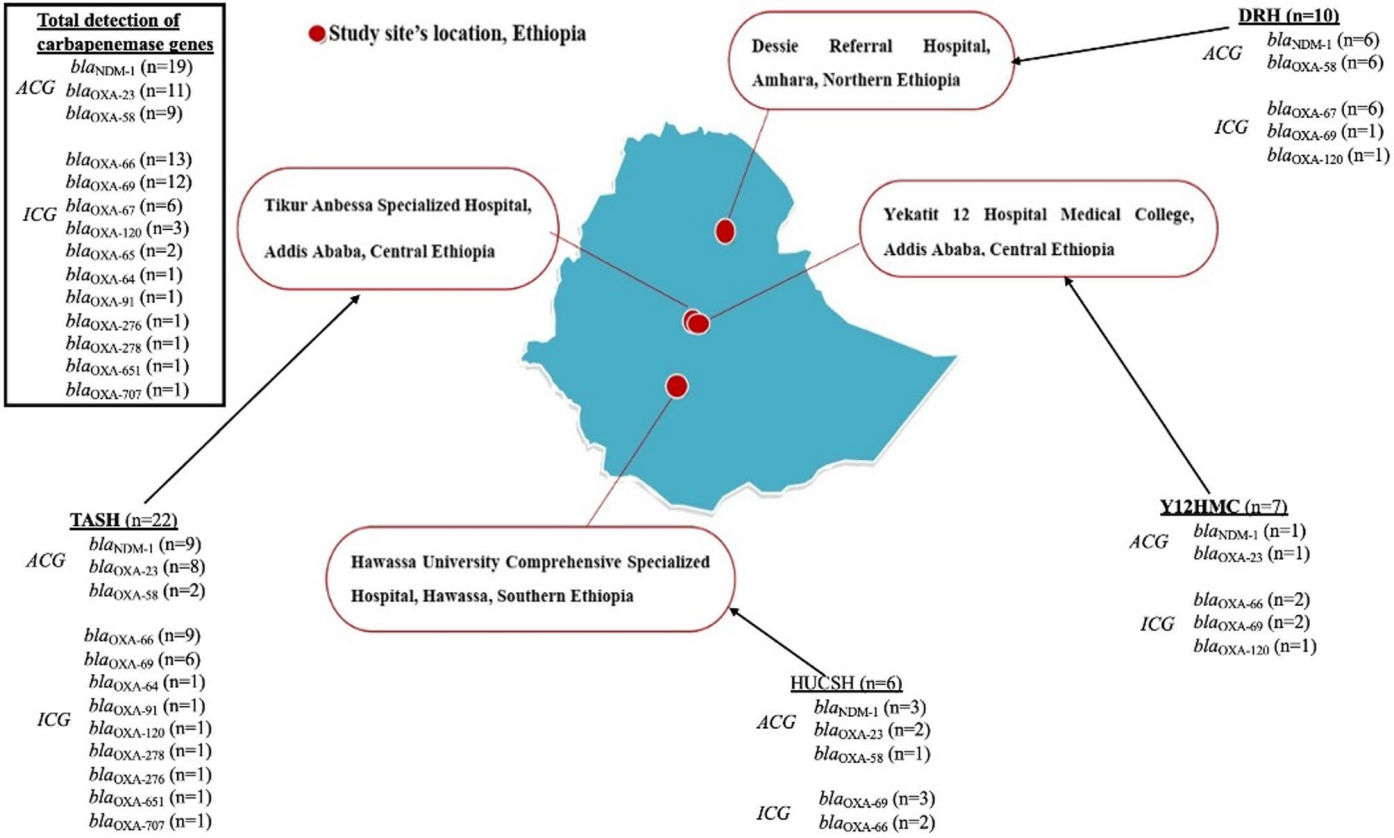
Frequencies and distributions of carbapenemase genes carried by Acinetobacter isolates (*n* = 45) identified at the four hospitals.TASH – Tikur Anbessa Specialized Hospital; Y12HMC – Yekatit 12 Specialized Hospital Medical College; DRH – Dessie Referral Hospital, HUCSH – Hawassa University Comprehensive Specialized Hospital; ACG - acquired carbapenemase genes; ICG - intrinsic carbapenemase genes.

The potential for carbapenemase production in *Acinetobacter* species was determined by genotypic detection of carbapenemase-encoding genes. Across the four study sites, variants of *bla*_NDM_ (*n* = 19) and *bla*_OXA_ (*n* = 20) carbapenemase genes were found in *A. baumannii* and other *Acinetobacter* species (Fig. 2). Among all *A. baumannii* isolates (*n* = 38) that underwent WGS, 47% (*n* = 18/38) carried *bla*_NDM−1,_ while 29% (*n* = 11/38) and 18.4% (*n* = 7/38) harbored *bla*_OXA−23_ and *bla*_OXA−58,_ respectively (Table 3). All *bla*_NDM−1_ harbouring *A. baumannii* isolates also carried either *bla*_OXA−23_ or *bla*_OXA−58_ concurrently (Table 3; Fig. 2). No instances of co-occurrence of *bla*_OXA−23_ and *bla*_OXA−58_ were observed. Among variants with variable frequencies, *bla*_NDM−1_ was the only *bla*_NDM_ variant circulating across wards at all four hospitals. *bla*_NDM−1_ was most commonly detected at TASH in the central part of the country, followed by DRH in the northern region (Fig. 2). All *bla*_NDM−1_ genes were found within an identical 8 kb region flanked by two transposase genes, suggesting that this region is a mobile genetic element. The 8 kb segment is commonly associated with plasmids, such as CP090865.1, but can also be part of the chromosome, as in CP130628.2. Among intrinsic chromosomally carried carbapenemase genes, *bla*_OXA−66_ and *bla*_OXA−69_ were the most frequently identified variants (Fig. 2).

**Table 3:** Frequency and distribution of carbapenemase genes carried by *Acinetobacter* species identified in four Ethiopian Hospitals.

Carbapenemase genes			*A. baumannii* (*N* = 38)				*A. johnsonii* (*N* = 1)	*A. schindleri* (*N* = 2)	*Acinetobacter species* (*N* = 1)	*A. nosocomialis* (*N* = 1)
			TASH (*n* = 18)	DRH (*n* = 8)	Y12HMC (*n* = 7)	HUCSH (*n* = 5)	TASH (*n* = 1)	TASH (*n* = 1)	TASH (*n* = 1)	HUCSH (*n* = 1)
*Acquired carbapenemase genes*		*bla*_NDM−1_	9(50)	6(75)	1(14)	2(40)	-	-	-	1(100)
		*bla*_OXA−23_	8(44)	-	1(14)	2(40)	-	-	-	-
		*bla*_OXA−58_	1(6)	6(75)	-	-	-	-	1(100)	1(100)
*Intrinsic carbapenemase genes*	*OXA-51-like*	*bla*_OXA−66_	9(50)	-	2(29)	2(40)	-	-	-	-
		*bla*_OXA−69_	6(33)	1(13)	2(29)	3(60)	-	-	-	-
		*bla*_OXA−67_	1(6)	6(75)	-	-	-	-	-	-
		*bla*_OXA−120_	1(6)	1(13)	1(14)	-	-	-	-	-
		*bla*_OXA−64_	1(6)	-	-	-	-	-	-	-
		*bla*_OXA−91_	1(6)	-	-	-	-	-	-	-
		*bla*_OXA−707_	1(6)	-	-	-	-	-	-	-
	*OXA-134-like*	*bla*_OXA−276_	1(6)	-	-	-	-	1(50)	-	-
		*bla*_OXA−278_	1(6)	-	-	-	-	1(50)	-	-
	*OXA-211-like*	*bla*_OXA−651_	1(6)	-	-	-	1(100)	-	-	-

TASH – Tikur Anbessa Specialized Hospital; Y12HMC – Yekatit 12 Specialized Hospital Medical College; DRH – Dessie Referral Hospital, HUCSH – Hawassa University Comprehensive Specialized Hospital; n- number of isolates; % - percentage per specific isolate.

**Fig. 3:**
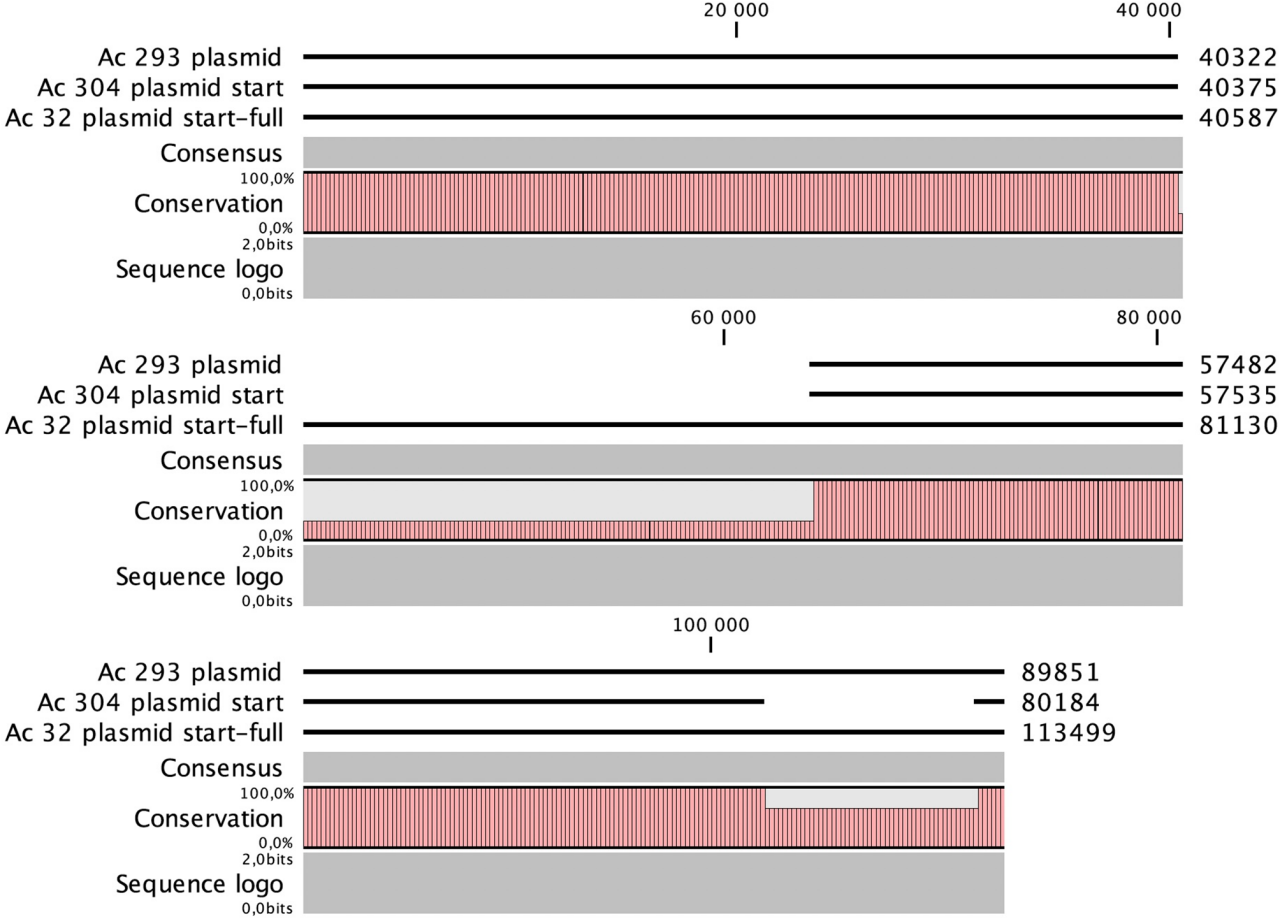
Plasmid sequencing illustration for selected Acinetobacter isolates. Note: Plasmids Ac 293 and Ac 304 are from Dessie, while Ac 32 is from Hawassa.

Nanopore sequencing of selected *Acinetobacter* isolates showed that *bla*_OXA−58_ and *bla*_NDM−1_ were co-located on plasmids of very similar sizes, ranging from 80 kb to 113 kb. Most of these isolates came from Dessie Referral Hospital. The largest plasmid (113 kb) was from an *A. nosocomialis* isolated at Hawassa Hospital. Significant portions of all plasmids were identical, and the size differences could be attributed to specific regions within the plasmid (Fig. 3).

The smallest plasmid, Ac 304, was missing a 9 kb segment compared to the other plasmids. This missing segment was flanked by transposases and contained the *floR* gene, which conferred chloramphenicol resistance via a transporter. The 113 kb Ac 32 plasmid included a 25 kb region with multiple genes encoding parts of the BREX phage exclusion system. Its replicon region was most similar to that of plasmid pNDM_SCLZS86 (CP090865.1), which also contains a BREX region. However, the fully sequenced plasmids differed significantly from the reference sequence and are listed in the NCBI database as SUB15589702.

In contrast, in isolates carrying both *bla*_OXA−23_ and *bla*_NDM−1_, the genes were chromosomally located. The entire section shows a high degree of similarity to the chromosomes of several *A. baumannii* isolates (e.g., JUNP405, AP031578.1). Notably, there was a very common linkage between *bla*_OXA−58_ and the two aminoglycoside resistance genes *aph(6)-1d* and *aph(3´´)-1b.* The only other example of this linkage is found in the chromosome of *A. pitti* (CP107289.1). All isolates carrying *bla*_OXA−23_ shared a typical multidrug resistance pattern, with 17 resistance genes, many of which encode aminoglycoside-modifying enzymes.

In addition to *A. baumannii*, though less often identified, other *Acinetobacter* species isolates also carried at least one carbapenemase gene, mostly *bla*_OXA_ variants (Table 3). One *A. nosocomialis* carried both *bla*_NDM−1_ and *bla*_OXA−58_ simultaneously. Among chromosomally encoded carbapenemase genes, *bla*_OXA−66_ (*n* = 13/38), *bla*_OXA−69_ (*n* = 12), and *bla*_OXA−67_ (*n* = 7/38) were often found in *A. baumannii*. *A. lwoffii* (*n* = 1), and *A. ursingii* (*n* = 1) did not carry carbapenemase-encoding genes.

### Genetic diversity and phylogenetic structure of *A. baumannii*

**Fig. 4:**
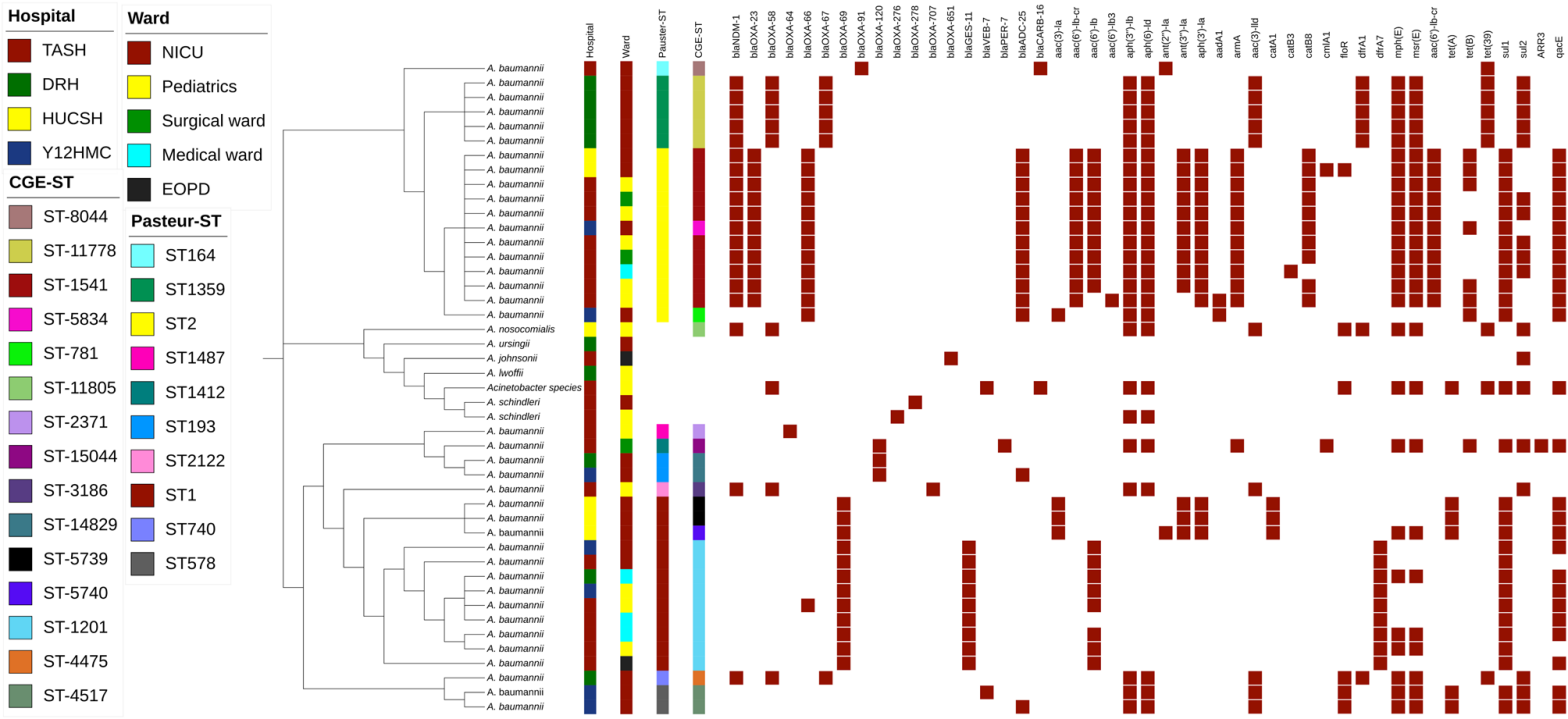
Phylogenetic relatedness, hospitals, isolation wards, sequence types (ST), and antimicrobial resistance genes encoded by Acinetobacter isolates. Note: Intrinsically carried carbapenemase genes are shown in the tree to see distribution in relation to isolates that carried them.

The core genome maximum-likelihood tree for 38 *A. baumannii* isolates showed that various clones circulated across the four hospitals. However, some of them were clonally identical (Fig. 4). Out of 18 *A. baumannii* isolates identified at TASH, a centrally located hospital, many were clonally clustered and related. However, some were very distinct (Fig. 4). Many belonged to ST1 and ST2, while the rest were of ST164, ST193, ST1412, and ST2122 (Fig. 4). Most *A. baumannii* clones isolated at TASH were circulating in its NICU and pediatric departments, although cases of different clones were found in other wards (Fig. 4). At Y12HMC, another centrally located hospital, all *A. baumannii* clones were unrelated except for two (Fig. 4). Nearly all *A. baumannii* clones identified at Y12HMC were isolated from the NICU (Fig. 4). The clones detected at Y12HMC included ST1, ST2, ST193, and ST578 (Fig. 4).

At HUCSH (from the south), all *A. baumannii* clones were identified in the NICU (Fig. 4) and belonged to ST1 and ST2 (Fig. 4). Notably, the ST1 isolates at HUCSH differed from other ST1 isolates in the cgMLST analysis. At DRH, all *A. baumannii* clones were detected in its NICU; most belonged to ST 1359, indicating a clonal outbreak in this ward. The other sequence types were diverse (Fig. 4), and all cases were found in both the NICU and medical ward (Fig. 4). The different *A. baumannii* clones at DRH included ST1, ST193, and ST740. Unlike other hospitals in the central (TASH and Y12HMC) and southern (HUCSH) regions, no cases of ST2 *A. baumannii* were identified at DRH in the northern part of the country.

### ESBLs and other antimicrobial resistance genes of *Acinetobacter* isolates

ESBL genes carried by *A. baumannii* were *bla*_VEB−7_, *bla*_GES−11_, and *bla*_PER−7,_ while *bla*_ADC−25_ was the only AmpC type identified. *bla*_ADC−25_ (*n* = 14) and *bla*_GES−11_ (*n* = 9) were the most common AmpC and ESBL genes, respectively, mainly found in different wards at TASH (Fig. 4). The *bla*_ADC−25_ carrying clone was ST2, *whereas bla*_GES−11_ was found in an ST1 *A. baumannii* clone (Fig. 4). *A. baumannii* isolates also carried non-ESBL beta-lactamase genes, though these were infrequent (Fig. 4), including *bla*_CARB−16_ and *bla*_SHV−89_. Most *A. baumannii* isolates also harbored several antimicrobial resistance (AMR) genes that inactivate aminoglycosides, tetracyclines, sulfonamides, macrolides, trimethoprim, and chloramphenicol (Fig. 4). Although less common, other *Acinetobacter* species possessed distinct AMR genes (Fig. 4). Only two *Acinetobacter* isolates (*A. lwoffii* and *A. ursingii*) lacked any antimicrobial resistance genes (Fig. 4).

## Discussion

Managing sepsis, a life-threatening emergency with a high mortality rate^3^, is always very challenging. This is especially true when caused by carbapenemase-producing *A. baumannii* (CP-Ab)^5^. In the current study, most *A. baumannii* isolates from sepsis patients in four hospitals were found to carry at least one carbapenemase gene. This indicates that CP-Ab is spreading as a dangerous pathogen in clinical environments. In resource-limited settings, outcomes of sepsis caused by CP-Ab are complicated by limited antimicrobial options, availability, and access, which can severely hinder patient recovery. Most *A. baumannii* cases were identified in patients hospitalized for at least 1 week or more than 4 weeks, or in those referred from other healthcare facilities, suggesting a hospital-acquired origin. Prolonged hospitalization has been recognized as a key risk factor for *A. baumannii* infection, consistent with prior studies.

Across all hospitals, multiple *A. baumannii* clones were identified, with ST1 and ST2 found in different wards, primarily in the NICU and the pediatric department. A clonal spread of *A. baumannii* in the NICU was reported from Brazil^16^. A study from Nepal^17^ also showed the circulation of ST1 and ST2 in its medical settings, indicating the global spread of these strains. In the current study, several additional *A. baumannii* STs were identified, showing the diversity of international clones circulating in the country. The presence of clonally related *A. baumannii* in specific wards, such as the NICU at DRH, suggests a potential clonal outbreak; however, only a few isolates shared ST and other characteristics necessary to define a clonal outbreak, so further investigation is needed to confirm this. The high proportion of referral cases among patients with positive cultures could increase diversity, as there is no documentation of the original referral site or prior hospitalizations.

The diversity of *A. baumannii* circulating in Ethiopian hospitals is a serious concern for all relevant stakeholders. The presence of different *A. baumannii* clones in the NICU and pediatrics departments greatly complicate sepsis management in neonates and children. Additionally, the detection of two distinct CP-Ab clones in NICUs across various hospitals highlights the importance of targeted infection prevention and sepsis management.

The high proportion of CP-Ab in this study was similar to that found in a study conducted in western Ethiopia on clinical cases other than sepsis or bloodstream infections^18^. This indicates that CP-Ab has spread across the country’s central, southern, northern, and western regions, which is concerning. Evidence of the global spread of CP-Ab in clinical settings has been confirmed in multiple studies^1,19–24^. A phenotypic antimicrobial resistance trend analysis revealed an increase in carbapenem-resistant *Acinetobacter species* within Ethiopian healthcare facilities^25^.

In the current study, *A. baumannii* isolates carried variants of acquired *bla*_OXA_ and *bla*_NDM−1_ carbapenemase genes simultaneously, making CP-Ab treatment difficult and worsening sepsis outcomes. Additionally, as seen in a previous study in western Ethiopia^18^, most *A. baumannii* carried intrinsically encoded OXA-51-like carbapenemase genes (*bla*_OXA−66_ and *bla*_OXA−69_), complicating the management of CP-Ab. All *A. baumannii* isolates with carbapenem resistance genes also harbored resistance genes against aminoglycosides and macrolides. Furthermore, the majority of CP-Ab strains possessed resistance determinants to quinolones, tetracyclines, sulphonamides, and disinfectants. The co-occurrence of carbapenem resistance genes with other AMR genes significantly limits remaining treatment options, making sepsis management caused by CP-Ab strains more difficult. The high number of these co-occurrences suggests the possibility of simultaneous transfer, for example, by plasmids. Nanopore sequencing revealed that the *bla*_OXA−58_ and *bla*_NDM−1_ genes were carried on 80–113 kb plasmids, as seen in selected *Acinetobacter* isolates. Plasmid carriage could facilitate the spread of carbapenemase genes in Ethiopian hospitals. Notably, five DRH isolates carried both *bla*_NDM−1_ and *bla*_OXA−58_. Four of these shared identical ST patterns, while the fifth differed, yet al.l five had identical resistance genes, including those in their immediate vicinity, indicating horizontal transfer. Similarly, *bla*_OXA−58_ was detected in several isolates across different STs. The *bla*_OXA−23_ gene was most commonly detected in ST2 isolates from various hospitals and wards. Nanopore sequencing showed that *bla*_OXA−23_ is chromosomal. In combination with multiple resistance genes, this suggests a clonal expansion of a successful *A. baumannii* strain. Consistent with a study conducted in Europe^26^, all 32 *Acinetobacter* isolates carrying *bla*_NDM−1_ and *bla*_OXA_ variants of carbapenemase genes were susceptible to cefiderocol, a finding previously reported^27^. However, MICs for most carbapenemase gene carriers ranged from 0. 0.5 to 2. 2.5 µg/ml, which are 10-fold higher than those for the most susceptible isolates.

Additionally, most *A. baumannii* identified in this study carried other AMR genes, reducing the antibiotic options for treating sepsis caused by multidrug-resistant *A. baumannii*. Some of these AMR genes were ESBLs, with *bla*_GES−11_ being the most common. The frequent detection of *bla*_GES−11_ matches findings from a study in western Ethiopia^17^, indicating its spread across different regions in the country. Carrying these ESBL genes allows the strains to inactivate cephalosporin antibiotics, limiting treatment choices. Furthermore, most *A. baumannii* isolates carried AMR genes conferring resistance to aminoglycosides, tetracyclines, sulphonamides, macrolides, trimethoprim, and chloramphenicol. This indicated that *A. baumannii* is highly resistant to most, if not all, available antibiotics, thereby increasing patients’ risk.

The high proportion of CP-Ab findings emphasizes the need for effective infection prevention and strict antimicrobial stewardship. While it is challenging to completely eliminate *A. baumannii* in clinical environments, consistent infection control efforts can help reduce its spread within healthcare facilities^21,28^.

Although infrequently identified, other *Acinetobacter* species that produce carbapenemases were detected. The emergence of non-*baumannii* MDR *Acinetobacter* species in sepsis patients has also been reported in several studies^12,29^. The detection of *bla*_NDM−1_-carrying *A. nosocomialis* in sepsis patients was documented in a previous study conducted in India^29^, indicating a global spread of these strains. This could further complicate the challenges posed by *Acinetobacter* species in clinical environments.

The main strength of this study was the use of whole-genome sequencing, which helped clarify how antimicrobial resistance develops in *A. baumannii* collected from four hospitals across the country’s central, southern, and northern regions. While choosing these four hospitals may introduce systematic bias, the results from these major referral hospitals, which serve a large population, could still be applicable to other healthcare settings in Ethiopia. However, the study’s limitations included an inability to identify the source of infection and a lack of patient outcome data to correlate with genomic data. Furthermore, using multiple blood culture bottles could have increased the chances of detecting more *Acinetobacter* species. Excluding patients who had taken antibiotics for 10 days might also impact the results, as these patients could harbor resistant microorganisms. Lastly, the lack of information about the origins of referral patients restricts the ability to track the clonal spread of *Acinetobacter* within and between wards in the selected hospitals.

## Conclusion

The study revealed a high rate of carbapenemase-producing *A. baumannii* among sepsis patients in hospitals across northern, southern, and central Ethiopia. The spread of *A. baumannii* strains carrying plasmid-mediated *bla*_OXA_ and *bla*_NDM−1_ genes poses a major public health issue. It is concerning that multiple *A. baumannii* clones with diverse antimicrobial resistance (AMR) genes are present in all hospitals. The high occurrence of multidrug-resistant *A. baumannii* in the NICU adds to these concerns. Additionally, the detection of other *Acinetobacter* species harboring carbapenemase genes complicates control efforts. These results underscore the urgent need for nationwide infection prevention and antibiotic stewardship initiatives.

## Supplementary Information

Below is the link to the electronic supplementary material.

Supplementary Material 1

## Data Availability

The genomic sequence data supporting this study were submitted to the National Center for Biotechnology Information (BioProject ID: PRJNA787062: SUB15589702 and SUB14785439).
