# Supplementary material for: High magnitude of carbapenemase-producing Acinetobacter baumannii in sepsis patients at Ethiopian referral hospitals: a whole genome analysis
Source: Sci Rep. 2026 Mar 18;16:14009. doi: 10.1038/s41598-026-44498-1 (PMC13133339; doi:10.1038/s41598-026-44498-1)
Supplement: Supplementary file 1 — Supplementary Material 1 [file 41598_2026_44498_MOESM1_ESM.docx]

Supplementary Table 1. Carbapenem resistance patterns and antimicrobial resistance level category of Acinetobacter species identified from sepsis patients in Ethiopian referral hospitals.

| *Acinetobacter species* | Carba disk diffusion testing | | Microbroth dilution- MIC testing | | E-test | | Resistance level category | | |
| --- | --- | --- | --- | --- | --- | --- | --- | --- | --- |
|  | R  n(%) | S  n(%) | R  n(%) | S  n(%) | R  n(%) | S  n(%) | MDR  n(%) | XDR n(%) | PDR n(%) |
| *A. baumannii (n=38)* | 30(79) | 8(21) | 30(79) | 8(21) | 35(92) | 3(8) | 36(95) | 7(18) | 6(16) |
| *A. johnsonii (n=1)* |  | 1(100) | 1(100) |  | 1(100) |  | 0(0) | 0(0) | 0(0) |
| *A. lwoffii (n=1)* |  | 1(100) |  | 1(100) |  | 1(100) | 0(0) | 0(0) | 0(0) |
| 1. *Nosocomialis (n=1)* | 1(100) |  | 1(100) |  | 1(100) |  | 1(100) | 0(0) | 0(0) |
| *A. schindleri (n=2)* |  | 2(100) | 1(50) |  | 2(100) |  | 0(0) | 0(0) | 0(0) |
| *Acinetobacter species (n=1)* | 1(100) |  | 1(100) |  | 1(100) |  | 1(100) | 0(0) | 0(0) |
| *A. ursingii (n=1)* |  | 1(100) |  |  |  | 1(100) | 1(100) | 0(0) | 0(0) |
| *Total (n=45)* | 32(71) | 13(29) | 34(76) | 9(24) | 40(89) | 5(11) | 39(87) | 7(16) | 6(13) |

*n- number of isolates; % - percentage per total isolate*
